# Revenge of the Microbes: How Bacterial Resistance Is Undermining the Antibiotic Miracle, 2nd Edition

**DOI:** 10.3201/eid3011.240228

**Published:** 2024-11

**Authors:** Isabella Caruso

**Affiliations:** University of Massachusetts Amherst, School of Public Health and Health Sciences, Amherst, Massachusetts, USA

**Keywords:** antimicrobial resistance, bacteria, microbiology, infectious disease, microbes

*Revenge of the Microbes: How Bacterial Resistance Is Undermining the Antibiotic Miracle*, 2nd edition, by Brenda A. Wilson and Brian T. Ho, is an intriguing and detailed narrative of the history of antibiotics, the mechanisms by which bacteria become resistant, and the spread of antibacterial resistance across the globe (Figure). The second edition comes at a critical time. While the attention of the general public, medical communities, and pharmaceutical companies is understandably focused on the development of vaccines and antivirals in response to the COVID-19 pandemic, this book shifts attention back to the ongoing crisis of antibiotic resistance.

**Figure:**
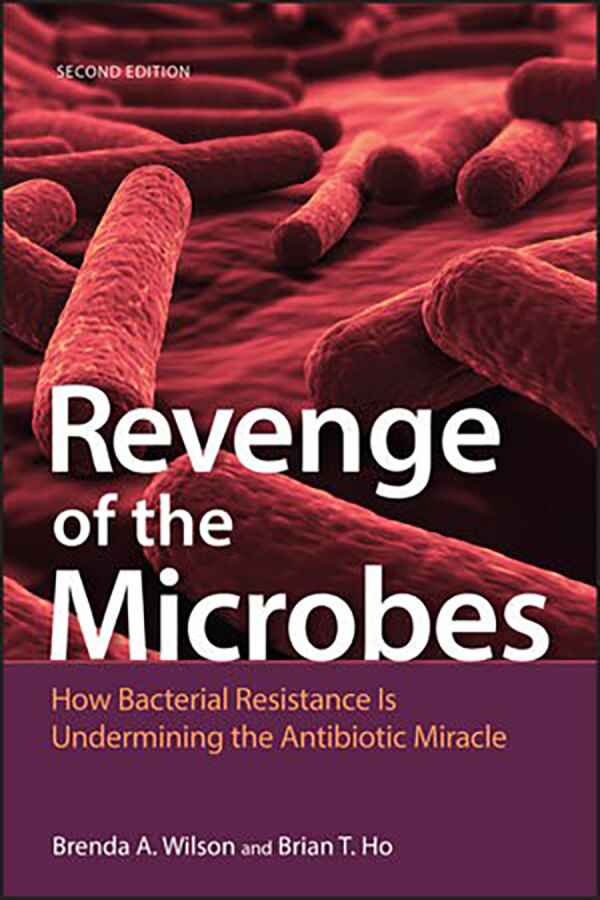
Revenge of the Microbes: How Bacterial Resistance Is Undermining the Antibiotic Miracle

The order of the chapters guides the reader seamlessly through the evolution of global antimicrobial resistance. The book begins with a brief overview of the history of antibiotics, diving into the groundbreaking antibiotic developments and discoveries. The chapters then explore the mechanisms by which bacteria gain resistance and how bacteria acquire resistance genes to existing antibiotics, providing the specific examples of avoparcin and fluroquinolones, which are of substantial public health interest.

The authors present readers with points to ponder, summarizing critical facts and discoveries while highlighting important unanswered questions at the conclusion of each chapter. That section provides an opportunity to pause, digest the material, and critically consider the potential impact of the “revenge of the microbes.”

The photographs, figures, and tables also enhance comprehension, especially for those who are new to the topic. For example, chapter 6 describes the processes in which bacteria become resistant to antibiotics, which include restricting the antibiotic’s access to the target, inactivating the antibiotic, and mutating the target itself. Figure 6.1 depicts a mechanism in which a membrane-embedded efflux pump actively removes tetracyclines, a class of antibiotics, in an easy-to-understand diagram. In addition, the included photograph of Anne Sheafe Miller, the first US patient to receive penicillin, emphasizes the authors’ claim that antibiotics are miracle drugs.

Wilson and Ho tactfully describe the intricacies of the antimicrobial resistance crisis, including discussions regarding antibacterial use in livestock, the economic pressures faced by pharmaceutical companies related to antibiotic development and assessment, and the importance of antibiotic stewardship in hospital settings. The book concludes by offering a multidisciplinary framework for key stakeholders and interest groups to tackle the antibiotic resistance crisis. For example, treating patients with antibiotic-resistant bacterial infections is costly and requires additional supplies, space, and equipment. Consequently, hospital administrators are allocating more resources to establish effective infection-control practices. The final chapter serves as a call to action, underscoring the necessity of participation from many sectors.

*Revenge of the Microbes *offers an insightful examination of a critical global public health threat. This text succinctly meets the objectives outlined in the preface, focusing on “bridging the informational divide by presenting a more holistic view of antibiotics and antibiotic resistance.” This book offers the opportunity for a general audience, including students, educators, scientists, medical professionals, and concerned citizens, to gain an appreciation for the successes of antibiotics and understand the intricacies of antibiotic use and stewardship.

